# Identifying geographic hot spots of reassortment in a multipartite plant virus

**DOI:** 10.1111/eva.12156

**Published:** 2014-04-09

**Authors:** Fiona R Savory, Varun Varma, Uma Ramakrishnan

**Affiliations:** National Centre for Biological Sciences, TATA Institute of Fundamental ResearchBangalore, India

**Keywords:** disease emergence, geographic hot spot, multipartite virus, reassortment

## Abstract

Reassortment between different species or strains plays a key role in the evolution of multipartite plant viruses and can have important epidemiological implications. Identifying geographic locations where reassortant lineages are most likely to emerge could be a valuable strategy for informing disease management and surveillance efforts. We developed a predictive framework to identify potential geographic hot spots of reassortment based upon spatially explicit analyses of genome constellation diversity. To demonstrate the utility of this approach, we examined spatial variation in the potential for reassortment among *Cardamom bushy dwarf virus* (CBDV; *Nanoviridae*, *Babuvirus*) isolates in Northeast India. Using sequence data corresponding to six discrete genome components for 163 CBDV isolates, a quantitative measure of genome constellation diversity was obtained for locations across the sampling region. Two key areas were identified where viruses with highly distinct genome constellations cocirculate, and these locations were designated as possible geographic hot spots of reassortment, where novel reassortant lineages could emerge. Our study demonstrates that the potential for reassortment can be spatially dependent in multipartite plant viruses and highlights the use of evolutionary analyses to identify locations which could be actively managed to facilitate the prevention of outbreaks involving novel reassortant strains.

## Introduction

Plant pathogens present a major challenge to food security and local economies as an estimated 10–16% of global food production is annually lost to disease (Strange and Scott 2005; Chakraborty and Newton 2011). Using evolutionary analyses to understand the processes that contribute to pathogen diversity and population structure could have important applications in plant disease management (Burdon and Thrall 2008; Prasanna et al. 2010; Acosta-Leal et al. 2011). For example, spatially explicit analyses which reveal how genetic variation is partitioned within and among plant pathogen populations can allow us to make predictions about future disease dynamics and to identify locations where novel pathogens may emerge (Burdon and Thrall 2008; Thrall et al. 2011). Here, we demonstrate how spatially explicit analyses of genetic diversity can be utilized to study geographic differences in the potential for reassortment in multipartite plant viruses.

Reassortment, or pseudo-recombination, occurs only in viruses with segmented genomes and involves the exchange of discrete genome components between different species or genetically distinct strains which coreplicate within the same host cell. This process generates hybrid progeny with novel combinations of genome components inherited from different parental viruses and may lead to the emergence of highly virulent strains (Hou and Gilbertson 1996; Pita et al. 2001; Gu et al. 2007; Chakraborty et al. 2008; Nelson et al. 2008; Chen et al. 2009) or facilitate adaptation to alternative hosts (Idris et al. 2008; Ince et al. 2013). Identifying geographic locations where reassortant lineages are most likely to emerge could be an important strategy to inform disease management and surveillance efforts (Pearce et al. 2009). However, relevant research emphasis has focused almost exclusively on viruses with segmented genomes that are important in the context of global health, such as influenza viruses (Koehler et al. 2008; Pearce et al. 2009; O'Keefe et al. 2010; Ramey et al. 2010; Wille et al. 2011; Barton et al. 2013; Fuller et al. 2013). There have been no explicit attempts to assess spatial variation in the potential for reassortment in multipartite plant viruses, such as begomoviruses (*Geminiviridae*), bromoviruses (*Bromoviridae*), nanoviruses (*Nanoviridae*), and tospoviruses (*Bunyaviridae*), which pose serious threats to the production of staple food crops and other economically important crops. Agricultural landscapes are often highly fragmented, containing a mosaic of patches of host, reservoir, and nonhost species, and this can lead to spatial genetic structure of plant pathogen populations (Plantegenest et al. 2007). Indeed, several multipartite plant viruses exhibit high levels of spatial population structure (Karan et al. 1994; Tsompana et al. 2005; Prasanna et al. 2010). As this creates geographic constraints on the ability of viruses to overlap and exchange genetic material (Prasanna et al. 2010; Martin et al. 2011a), population structure may lead to geographic differences in the potential for reassortment between genetically distinct strains.

Given that reassortment generates novel combinations of genome components derived from different species or strains, the diversity of combinations that are observed in a population (hereafter referred to as genome constellation diversity) can provide an indication of the frequency of past reassortment events (Dugan et al. 2008). Analyses of genome constellation diversity could also be used to make predictions about the occurrence of future reassortment events. This is because the probability that coinfections will involve viruses with the potential to reassort is determined by the diversity of genotypes with distinct genome constellations which cocirculate in a given location (Barton et al. 2013). Assuming that disease incidence is sufficiently high for coinfections to occur, reassortment is more likely to occur in locations where viruses exhibit a variety of genome constellations, than in locations where the majority of viruses are genetically identical or highly homogeneous in all genome components. Identifying geographic locations where genome constellation diversity is conducive to reassortment could thus be an informative approach used to target areas for increased surveillance and control of multipartite plant viruses or animal viruses with segmented genomes.

For reassortment to be detectable and relevant in an evolutionary and epidemiological context, hybrid progeny must be viable and exhibit little or no fitness deficit relative to other genotypes which cocirculate in a population. Therefore, when genome components from different parental viruses are reassembled, they must be able to function as efficiently as they did within the genomic backgrounds in which they evolved (Martin et al. 2011a). High levels of nucleotide sequence dissimilarity between parental viruses can constrain the fitness of hybrid progeny by disrupting coevolved intragenome interactions (Martin et al. 2005, 2011b; Escriu et al. 2007; Lefeuvre et al. 2007; Rokyta and Wichman 2009). Although the extent to which hybrid fitness declines with increasing genetic distance between parental strains can vary according to the genomic region which is exchanged, genetic exchange among plant viruses rarely yields viable progeny when levels of parental nucleotide sequence identity are lower than 90% (Martin et al. 2005; Escriu et al. 2007; Lefeuvre et al. 2007). However, reassortment between viruses which belong to the same species could often generate viable hybrids if the genome components which are inherited from different parental genotypes are functionally compatible (Grigoras et al. 2014). Surveillance and control efforts should thus be focused in locations where sequence divergence among potential parental viruses is sufficiently high for reassortment to yield hybrid progeny with novel phenotypes, yet sufficiently low for intragenome interactions to be preserved.

We developed a predictive framework to identify potential geographic hot spots of reassortment among *Cardamom bushy dwarf virus* (CBDV, *Nanoviridae*, *Babuvirus*) genotypes in Northeast India. CBDV is an aphid-borne nanovirus within the *Babuvirus* genus (Mandal et al. 2004, 2013). Nanoviruses have multipartite genomes, consisting of up to 12 individually encapsidated, circular, single-stranded DNA (ssDNA) genome components (Mandal 2010), and are known to undergo reassortment (Timchenko et al. 2000; Hu et al. 2007; Fu et al. 2009; Hyder et al. 2011; Stainton et al. 2012; Grigoras et al. 2014; Savory and Ramakrishnan 2014). CBDV infects large cardamom, *Amomum subulatum*, a crop of considerable economic importance in sub-Himalayan regions of Northeast India, Nepal, and Bhutan. It is the causal agent of ‘foorkey’ disease, which is characterized by excessive sprouting of dwarf tillers, reduced yield and mortality, and severely constrains large cardamom production (Mandal et al. 2013). Importantly, CBDV isolates from Northeast India exhibit moderately low levels of genetic diversity (Savory and Ramakrishnan 2014). This suggests that reassortment could generate viable hybrid lineages, as levels of nucleotide sequence identity between potential parental viruses may be sufficiently high for DNA–protein and protein–protein interaction networks to be preserved. Indeed, reassortment appears to have played an important role in the evolutionary dynamics of CBDV in the past (Savory and Ramakrishnan 2014). Using a phylogenetic approach to assign viruses to specific genome constellations, we first assessed the potential for reassortment by investigating whether isolates with distinct genome constellations cocirculate in the same geographic localities. We then made predictions about where reassortment is most likely to occur based upon geographic differences in genome constellation diversity.

## Methods and materials

### Sample collection and study area

Leaf samples were collected in 2011 and 2012 from CBDV-infected plants at a range of locations and altitudes throughout the state of Sikkim and the Darjeeling district of West Bengal, Northeast India (Table S1). Samples were dried with silica gel and were maintained in Ziplock bags until DNA extraction. Large cardamom plants are cultivated across an elevation gradient ranging from approximately 500 to 2000 m above sea level. Due to the rugged topology of the landscape, there are limited continuous tracts in which climatic conditions are suitable for large cardamom cultivation, and large-scale tea plantations dominate the landscape in Darjeeling. These factors have resulted in a highly patchy distribution of large cardamom plantations. As it was not possible to sample with equal intensity across space, this is reflected in our sampling distribution.

### DNA extraction and PCR amplification of CBDV genome components

Approximately 100 mg of leaf tissue from 163 infected plant samples was ground to a fine paste with a mortar and pestle using liquid nitrogen. Total genomic plant DNA and viral DNA were then simultaneously extracted using a Nucleospin Kit (Genetix Biotech Asia Pvt. Ltd., Bangalore, Karnataka, India). Full-length sequences corresponding to six discrete CBDV genome components (DNA-R, DNA-U3, DNA-S, DNA-M, DNA-C, and DNA-N; each approximately 1.1 kb in length) were directly obtained from these DNA extractions using previously described primers (Savory and Ramakrishnan 2014). We refer to the virus sample obtained from a single infected plant as an isolate. However, in the event that any of the plants were coinfected by multiple CBDV genotypes, the sequences obtained for the corresponding isolate would represent consensus sequences. PCRs were performed in 10 μL reaction volumes, containing 3.4 μL H_2_O, 5 μL Multiplex PCR Master Mix (Qiagen), 0.3 μL of each primer, and 1 μL DNA template. PCR protocols were identical for all six genome components and involved initial denaturation for 15 min at 95°C, 34 cycles of denaturation for 30 s at 94°C, annealing for 1 min at 55°C, and extension for 1 min at 72°C, and then a final extension period of 10 min at 72°C. Sequencing was performed using a 3130xl Genetic Analyzer from Applied Biosystems (Life Technologies, Carlsbad, CA, USA). Sequences were aligned and edited using MEGA version 5.05 (Tamura et al. 2011). GenBank accession numbers are as follows: KF710463 – KF710625 (DNA-R), KF710626 – KF710788 (DNA-U3), KF710789 – KF710951 (DNA-S), KF10952 – KF11114 (DNA-M), KF711115 – KF711277 (DNA-C), and KF11278 – KF711440 (DNA-N; Table S1).

### Phylogenetic analyses

Bayesian phylogenies were reconstructed for each genome component using a Markov chain Monte Carlo (MCMC) method implemented in BEAST version 1.7.1 (Drummond et al. 2012). Prior to the analyses, a region of 46 bp in length was removed from the 3′ end of the DNA-R sequences of 12 isolates (GenBank accession numbers: KF10478, KF10542, KF10563, KF10577 – KF10583, KF10593, KF10605), and a region of approximately 120 bp in length was removed from the 3′ end of the DNA-U3 sequences of 16 isolates (GenBank accession numbers: KF10630, KF10632, KF10672, KF10673, KF10693, KF10698, KF10712, KF10714, KF10716, KF10718, KF10719, KF10722, KF10733, KF10760, KF10775, KF10781) because intercomponent recombination was considered to confound the phylogenetic inference. The recombinants were identified by applying recombination detection tests implemented in RDP4 (Martin et al. 2010) to an alignment of the sequences obtained for all six genome components (Savory and Ramakrishnan 2014). For all genome components, we applied a general time reversible (GTR) model of nucleotide substitution with gamma-distributed rate heterogeneity across sites and an uncorrelated, relaxed, lognormal molecular clock model (Drummond et al. 2006). The GTR model of nucleotide substitution was selected using jModelTest 2 (Darriba et al. 2012). Phylogenetic analyses were run twice for each component. For the DNA-R, DNA-S, and DNA-C datasets, each analysis was run for 20 000 000 MCMC generations, with trees and parameters being sampled every 1000 generations. For the DNA-U3, DNA-M, and DNA-N datasets, each analysis was run for 100 000 000, 40 000 000, and 50 000 000 MCMC generations, respectively, and trees and parameters were sampled every 5000, 2000, and 2500 generations, respectively. Convergence of the MCMC chains for each genome component was confirmed using TRACER version 1.5 (Rambaut and Drummond 2007). The posterior distribution of trees from the two independent runs for each genome component was combined using LogCombiner version 1.7.1 (Drummond et al. 2012) after removal of 20% burn-in. Maximum clade credibility (MCC) trees were obtained from the posterior distribution of trees for each genome component using TreeAnnotator version 1.7.1 (Drummond et al. 2012), and trees were visualized using FigTree version 1.3.1 (Rambaut 2009).

### Phylogenetic assignment and nonmetric multidimensional scaling

**Figure 1:**
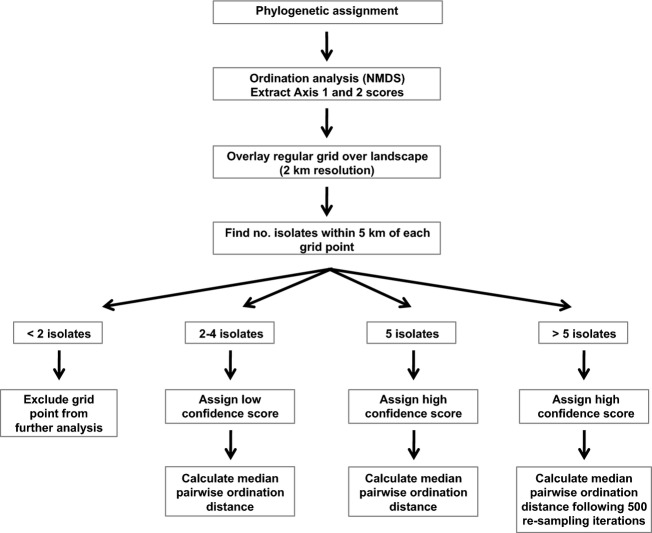
Flow chart of methods.

Major and minor clades that were supported by Bayesian posterior probabilities of ≥0.8 were identified following visualization of the phylogenetic tree for each genome component. For all genome components, isolates were assigned to one of two major clades, and for three components (DNA-S, DNA-M, and DNA-N), isolates were further divided into minor clades (Figures S1–S6). Genetic distances within and between clades were calculated according to the mean number of pairwise nucleotide substitutions per site (Nei 1987) using DnaSP version 5 (Rozas and Rozas 1999; Table S2). Genome constellations were characterized for each isolate based upon the specific combination of clades to which they were inferred to belong (Fig. 1). Viruses which were assigned to the same genome constellations thus had shared evolutionary histories and high levels of sequence identity for all genome components (Figures S1–S6; Table S2). Nonmetric multidimensional scaling (NMDS) with Bray–Curtis dissimilarities was used to collapse the clade membership information to a two-dimensional dataset, and the genome constellation of each isolate was subsequently described using NMDS scores 1 and 2 (Fig. 1). This allowed us to obtain an index of dissimilarity for each pair of isolates based upon the distance between their genome constellations in two-dimensional ordination space. NMDS was implemented using the Vegan package (Oksanen et al. 2008) in R version 2.15 (R Development Core Team 2013) and was repeated iteratively to minimize the stress (a stress value of 0.062 was achieved), the degree of mismatch between the calculated distances between each pair of isolates and their pairwise distances in ordination space.

### Spatial variation in the potential for reassortment

A regular grid of points of 2 km increments was generated to cover the extent of the study area using Python version 2.7.3 (http://www.python.org; Fig. 1). Geographic distances from each grid point to each sampling location were calculated, and then, grid points which had <2 CBDV isolates collected within a 5 km radius were discarded from the analysis (Fig. 1). For each remaining grid point, the median pairwise ordination distance among all CBDV isolates collected within a 5 km radius was calculated to obtain a quantitative measure of genome constellation diversity for that location (Fig. 1). We considered 5 km to be an appropriate spatial scale at which to measure genome constellation diversity based on an assumption that vector-mediated and/or human-mediated dispersal of CBDV isolates could conceivably occur across this distance. Two approaches were used to account for unequal sampling intensity across the landscape (Fig. 1). Firstly, grid points were assigned a low confidence score if 2–4 samples had been collected within a 5 km radius and a high confidence score if five or more samples had been collected within a 5 km radius. Secondly, a resampling approach was implemented to recalculate the median pairwise ordination distances for all grid points for which more than five isolates had been collected within a 5 km radius. Median pairwise ordination distances were recalculated 500 times for each of these grid points by repeatedly resampling a subset of five isolates without replacement. The overall median of the median pairwise ordination distances for a given grid point was then used as the final measure of genome constellation diversity for that location. Spatial variation in genome constellation diversity was visually represented using QGIS version 2.2 (Quantum GIS Development Team 2014; http://www.qgis.org).

### Analysis of population structure

Population structure was assessed using STRUCTURE version 2.3.3 (Pritchard et al. 2000), which implements a model-based Bayesian clustering algorithm to identify groups of genetically similar individuals and divergent populations. The analysis was performed on a single nucleotide polymorphism (SNP) dataset containing 967 polymorphic sites from across the six genome components that were observed to vary in at least two isolates (singleton SNPs were not considered). To estimate the number of CBDV subpopulations, *K* values (the number of populations) were allowed to vary from 1 to 5, and five independent runs for each *K* value were implemented. Initial runs with different burn-in and parameter estimation periods were compared to ensure the reliability of posterior probability estimates. The final run included an initial burn-in period of 100 000 iterations and a parameter estimation period of 100 000 iterations. The admixture model with correlated allele frequencies between populations was selected to account for individuals with mixed ancestry and to allow for similar allele frequencies between populations. This model is appropriate for viruses which exhibit high rates of genetic exchange and migration (Prasanna et al. 2010). The optimum number of subpopulations was determined using the ΔK method (Evanno et al. 2005), which was implemented in STRUCTURE HARVESTER (Earl and vonHoldt 2012). *Q* values (indicating the proportion of ancestry from each of *K* clusters) from the five independent runs for the optimal *K* value were compiled using CLUMPP (Jakobsson and Rosenberg 2007) and visualized using Distruct (Rosenberg 2004) and then were used to assign individuals to inferred subpopulations. Individuals were considered to be admixed if they could be assigned to two or more subpopulations with *Q* values of 0.15 or above. The clustering algorithm which is implemented in STRUCTURE assumes that polymorphic sites in the data being assessed do not display strong levels of linkage disequilibrium. Given that both recombination and reassortment occur in nanoviruses (Hu et al. 2007; Fu et al. 2009; Hyder et al. 2011; Stainton et al. 2012; Wang et al. 2013; Grigoras et al. 2014; Savory and Ramakrishnan 2014) and that these processes are likely to break down linkage between polymorphic sites on the same genome component and on different genome components respectively, we expected levels of linkage disequilibrium to be low.

To verify the population divisions that were inferred by STRUCTURE, an analysis of molecular variance (amova) was implemented in ARLEQUIN version 3.5 (Excoffier and Lischer 2010) after removal of admixed individuals. amova tests the partitioning of molecular variation among predefined subpopulations and yields fixation indices (*F*_ST_ values) which describe the extent of genetic differentiation for a given level of population subdivision. The significance of the *F*_ST_ value was assessed using a nonparametric permutation test with 1000 iterations (Weir and Cockerham 1984).

## Results

### Genome constellation diversity

Six discrete genome components were sequenced for 163 CBDV isolates collected throughout Sikkim and the Darjeeling district of West Bengal, Northeast India. These components included DNA-R, which encodes the CBDV master replication protein, DNA-U3, which has an unknown function, DNA-S, which encodes the CBDV coat protein, DNA-M, which encodes the CBDV movement protein, DNA-C, which encodes the CBDV cell cycle link protein, and DNA-N, which encodes the CBDV nuclear shuttle protein (GenBank accession numbers are listed in Table S1). Following phylogenetic assignment of each isolate to a specific combination of major and minor clades, 34 unique genome constellations were observed. The frequencies of these different constellations were highly skewed (Figure S7), suggesting that there may be fitness advantages associated with specific genome constellations that are perhaps environment-dependent. Importantly, the apparent stability of certain genome constellations implies that at least in some locations, a high proportion of viral isolates are likely to share the same constellation.

### Geographic overlap of isolates with distinct genome constellations

**Figure 2:**
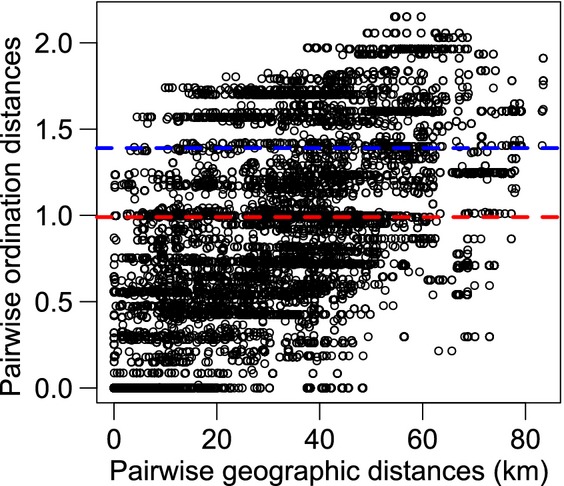
Pairwise ordination distances versus pairwise geographic distances. The red dashed line corresponds to the median pairwise ordination distance, and the blue dashed line corresponds to the upper quartile pairwise ordination distance when pairwise comparisons for all *Cardamom bushy dwarf virus* isolates were considered.

To ascertain whether epidemiologically relevant reassortment events are likely to occur among CBDV isolates, we assessed whether isolates with distinct genome constellations cocirculate in the same geographic localities. Pairwise ordination distances (reflecting the level of dissimilarity between the genome constellations of each pair of isolates) were positively associated with pairwise geographic distances (*R*^2^ = 0.29, *P* < 0.001), demonstrating that genome constellations get increasingly dissimilar with greater geographic distances between isolates (Fig. 2). Nevertheless, isolates with distinct genome constellations were observed at relatively small spatial scales at which dispersal may readily occur. For instance, at the scale of a single plantation or adjacent small plantations (0–0.5 km), pairwise ordination distances ranged from 0 (identical genome constellations) to 1.25, and at slightly larger spatial scales (0–5.0 km), pairwise ordination distances ranged from 0 to 1.6. Notably, the maximum pairwise ordination distances observed at these scales exceed the median (0.99) and upper quartile (1.39) pairwise ordination distances, respectively, when pairwise comparisons among all 163 CBDV isolates were considered (Fig. 2). This suggests that a considerable proportion of the total genome constellation diversity that is observed across the entire sampling region can be present in the same geographic locality. Assuming that dispersal readily occurs across short distances (e.g. ≤5.0 km) and that disease incidence is sufficiently high for coinfections to occur, these results indicate that there is high potential for reassortment between CBDV isolates with distinct genome constellations.

### Identifying geographic hot spots of reassortment

**Figure 3:**
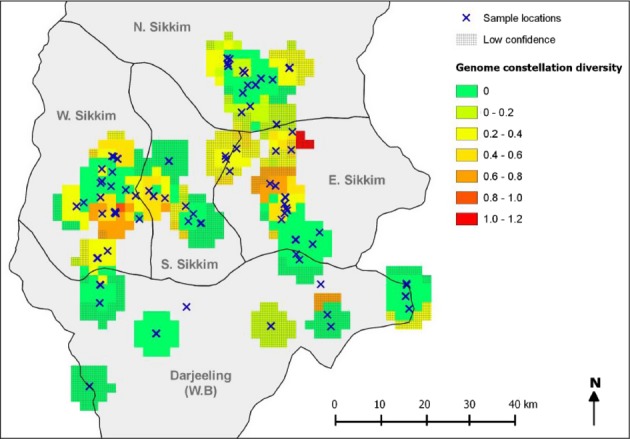
Spatial variation in genome constellation diversity in the North (N), East (E), South (S), and West (W) districts of Sikkim and the Darjeeling district of West Bengal (W.B). Genome constellation diversity was calculated using the median pairwise ordination distance for all isolates collected within a 5 km radius of each grid point. Shaded areas represent locations where grid points were assigned a low confidence score (<5 samples).

To examine geographic differences in the potential for reassortment, we considered how genome constellation diversity changes across space, using the median pairwise ordination distance among all isolates collected within a 5 km radius as the measure of genome constellation diversity for a given location. Considerable variation in genome constellation diversity was observed across the sampling region (Fig. 3). This variation does not reflect geographic differences in sampling intensity because no clear relationship was observed between median pairwise ordination distances and the number of viral isolates considered (correlation coefficient < 0.1; Figure S8). Therefore, our results imply that the potential for reassortment is spatially dependent on CBDV.

The majority of locations, including those which had been assigned a high confidence score (five or more samples collected within a 5 km radius), had relatively low levels of genome constellation diversity (Fig. 3). Although distinct genome constellations could be introduced into these locations by migration, the current lack of diversity suggests that novel reassortant lineages are unlikely to emerge in these areas. Locations which harbor isolates with a relatively high diversity of genome constellations were observed in specific areas of West Sikkim and East Sikkim (Fig. 3). These locations had been assigned a high confidence score and had median pairwise ordination distances ranging between 0.6 and 1.0. The high levels of genome constellation diversity observed among isolates in these areas are broadly comparable to the overall level of genome constellation diversity observed when pairwise comparisons among all 163 CBDV isolates were considered (median pairwise ordination distances for all isolates = 0.99). The latter represents a level of genome constellation dissimilarity that could be expected between two isolates that are selected at random from any locations across the sampling region. Therefore, the high levels of diversity observed in specific areas of West Sikkim and East Sikkim are analogous to those which may occur under a scenario in which CBDV isolates are not spatially structured. As there is a high chance that two isolates which coinfect the same host plant will have distinct genome constellations under such a scenario, we designated the locations as possible geographic hot spots of reassortment.

**Figure 4:**
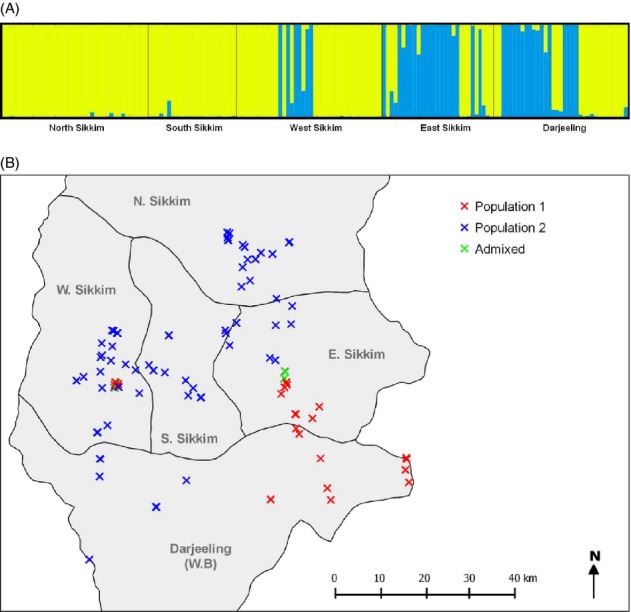
(A) Clustering of isolates based on a Bayesian analysis of population structure implemented in STRUCTURE version 2.3.3 (optimal number of populations: *K* = 2). Vertical bars correspond to individual *Cardamom bushy dwarf virus* isolates. Isolates were grouped according to their districts of origin. The two inferred populations are indicated by different colors, and when both colors are present within an individual bar, this indicates admixture. (B) Population membership and admixture of *Cardamom bushy dwarf virus* isolates collected in the North (N), East (E), South (S), and West (W) districts of Sikkim and the Darjeeling district of West Bengal (W.B). The geographic coordinates of isolates in the area of the predicted reassortment hot spot in West Sikkim have been jittered to facilitate visualization of admixed isolates and isolates which were assigned to different populations.

We expected that the areas which exhibit the highest levels of genome constellation diversity should be situated in locations where geographically defined subpopulations overlap. An analysis of population structure which was implemented using STRUCTURE version 2.3.3 (Pritchard et al. 2000) supported the existence of two geographically stratified subpopulations of CBDV isolates (Fig. 4A,B). Population subdivision was verified by an amova test which yielded a highly significant *F*_ST_ statistic (*F*_ST_ = 0.31, *P* < 0.001). While 116 of the isolates could be assigned to one subpopulation, 39 were assigned to the other. The remaining eight isolates were inferred to be admixed, and, of these, six had been collected in the vicinity of the predicted hot spots of reassortment in East Sikkim (four isolates) and West Sikkim (two isolates). The distributions of the two CBDV subpopulations supported our expectation that spatial overlap underlies the high genome constellation diversity observed in the East Sikkim reassortment hot spot (Fig. 4B). However, the high genome constellation diversity observed in the West Sikkim reassortment hot spot appears to have arisen due to past migration and subsequent admixture (Fig. 4B).

## Discussion

Targeted management strategies which reduce the potential for reassortment in specific locations by controlling disease incidence could facilitate the prevention of outbreaks involving novel reassortant strains. However, few studies have considered how the potential for future reassortment events can vary across space (Pearce et al. 2009; Fuller et al. 2013). We have demonstrated that the potential for reassortment can be spatially dependent in multipartite plant viruses and have introduced a framework which can be used to make predictions about where novel reassortant lineages are most likely to emerge. Additional information could be incorporated to refine these predictions if available. In particular, data regarding disease incidence, viral load, and/or the duration of infectivity could be highly informative, as these factors influence the frequencies at which genetically distinct strains coreplicate within the same host cells (Martin et al. 2011a).

The majority of locations throughout the sampling region appear to contain CBDV isolates with a relatively low level of genome constellation diversity, indicating that a high proportion of isolates in these locations share the same genome constellation. Although we did not account for reassortment among closely related isolates which were assigned to the same clades for all genome components, genetic exchange between such viruses may be irrelevant in an evolutionary and epidemiological context because the phenotypes of hybrid progeny are likely to be the same as, or highly similar to, those of the parental genotypes. Our analysis highlighted two key areas which harbor CBDV isolates with a relatively high diversity of genome constellations. As these represent locations where novel reassortant lineages may emerge if coinfections occur, the information which we have generated in this study could yield guidelines for policymakers and landowners involved in the formulation of large cardamom disease management strategies.

Our assessment of spatial population genetic structure indicated that the high levels of genome constellation diversity can be attributed to the overlap of isolates from distinct subpopulations (East Sikkim) and to one or more past migration events from one subpopulation to another (West Sikkim). CBDV is naturally transmitted by the aphids *Pentalonia nigronervosa* (Varma and Capoor 1964) and *Micromyzus kalimpongensis* (Basu and Ganguly 1968). Topological features of the landscape and/or environmental variables associated with elevation may indirectly influence patterns of CBDV movement and lead to the observed patterns of population structure by affecting aphid dispersal. However, it has been suggested that human transport of infected tillers is the primary mechanism underlying CBDV movement over long distances (Nair 2011). Therefore, it is perhaps more likely that socioeconomic factors and local transport networks underlie the observed patterns of spatial population genetic structure. Given that the reassortment hot spots that were predicted by our analysis could also be detected as high-risk areas using well-established population genetic analyses, such as implemented in STRUCTURE, these approaches could feasibly be used to identify areas where control strategies should be implemented. However, such analyses can be problematic as they do not always yield consistent results across multiple runs and are sensitive to variation in sample size (Kalinowksi 2011). The approach that we have developed does not rely on accurate inference of populations or subpopulations and explicitly quantifies a measure of genome constellation diversity for each location while accounting for differences in sampling intensity across space.

As hybrid viability will largely be determined by the genomic locations and types of nucleotide differences that exist between parental viruses, one cannot make accurate predictions about the fitness of reassortant lineages simply based upon overall levels of parental sequence divergence. Indeed, in laboratory-constructed *Maize streak virus* (MSV, *Geminiviridae*, *Mastrevirus*) recombinants, the level of parental sequence dissimilarity that can be tolerated varies according to the number of intragenome interactions in which the exchanged genomic regions are known to be involved (Martin et al. 2005). Patterns of reassortment are nonrandom in CBDV (Savory and Ramakrishnan 2014) and other nanoviruses (Stainton et al. 2012; Grigoras et al. 2014), suggesting that different genome components vary in their abilities to function efficiently when introduced into foreign genomic backgrounds. Infectious cloned genomes have recently been produced for several nanovirus species, and these may allow the fitness consequences of exchanging specific genome components to be experimentally assessed (Grigoras et al. 2014). Such information could be used to refine spatial predictions of reassortment hot spots, for instance by incorporating a weighting parameter within the framework which penalizes parental sequence divergence above a threshold level that can be specified independently for each genome component.

Focusing surveillance and control efforts in locations where cocirculating viruses exhibit a variety of genome constellations may be effective even in the absence of detailed information regarding possible fitness costs associated with reassortment. This is because reassortment between parental viruses with highly distinct genome constellations would enable expansive regions of sequence space to be explored. For a multipartite virus with six discrete genome components, reassortment between two parents which differ in two components could yield only four (2^2^) possible genome constellations, but reassortment between parents which differ in all six components could yield 64 (2^6^) possible genome constellations (including the parental genomes and chimeric genomes containing components derived from each parent). Although many of the reassortant lineages may be selected against, some may be viable and some may even confer a selective advantage over other genotypes in the population.

The framework that we developed in this study is based upon current levels of genome constellation diversity. Several studies have demonstrated that genetic diversity can remain stable over time in plant virus populations (García-Arenal et al. 2001). However, genetic diversity can be highly dynamic in some virus populations (Ghedin et al. 2005; Rambaut et al. 2008). Transient events, such as selective sweeps or population bottlenecks, can temporarily reduce genetic diversity (Li and Roossinck 2004; Rambaut et al. 2008), and migration can introduce variation into a subpopulation that was previously genetically homogeneous. Nevertheless, we believe that the approach we have introduced could have applications in plant disease management as it may allow surveillance and control efforts to be implemented effectively for a variety of multipartite plant viruses in strategic locations, such as areas where subpopulations overlap. These may represent high-risk locations, where reassortment could facilitate evolutionary innovation and lead to the emergence of novel lineages with the capacity to evade host immunity, expand host ranges, or overcome treatment interventions.

## Data archiving statement

Sequences that were used in this study are available from GenBank, and accession numbers are provided in the supplementary materials.

## Supporting Information

**Figure S1.** DNA-R phylogeny.

**Figure S2.** DNA-U3 phylogeny.

**Figure S3.** DNA-S phylogeny.

**Figure S4.** DNA-M phylogeny.

**Figure S5.** DNA-C phylogeny.

**Figure S6.** DNA-N phylogeny.

**Figure S7.** Frequency distribution of unique genome constellations.

**Figure S8.** Median pairwise ordination distance (genome constellation diversity) versus number of samples collected within a 5 km radius.

**Table S1.** List of CBDV isolates with corresponding districts, geographic coordinates, elevations, and GenBank accession numbers for each genome component.

**Table S2.** Genetic distances within and between clades for each genome component.
