# Supplementary material for: Identifying geographic hot spots of reassortment in a multipartite plant virus
Source: Evol Appl. 2014 Apr 9;7(5):569–79. doi: 10.1111/eva.12156 (PMC4055178; doi:10.1111/eva.12156)
Supplement: Supplementary file 1 [file eva0007-0569-SD1.pdf]

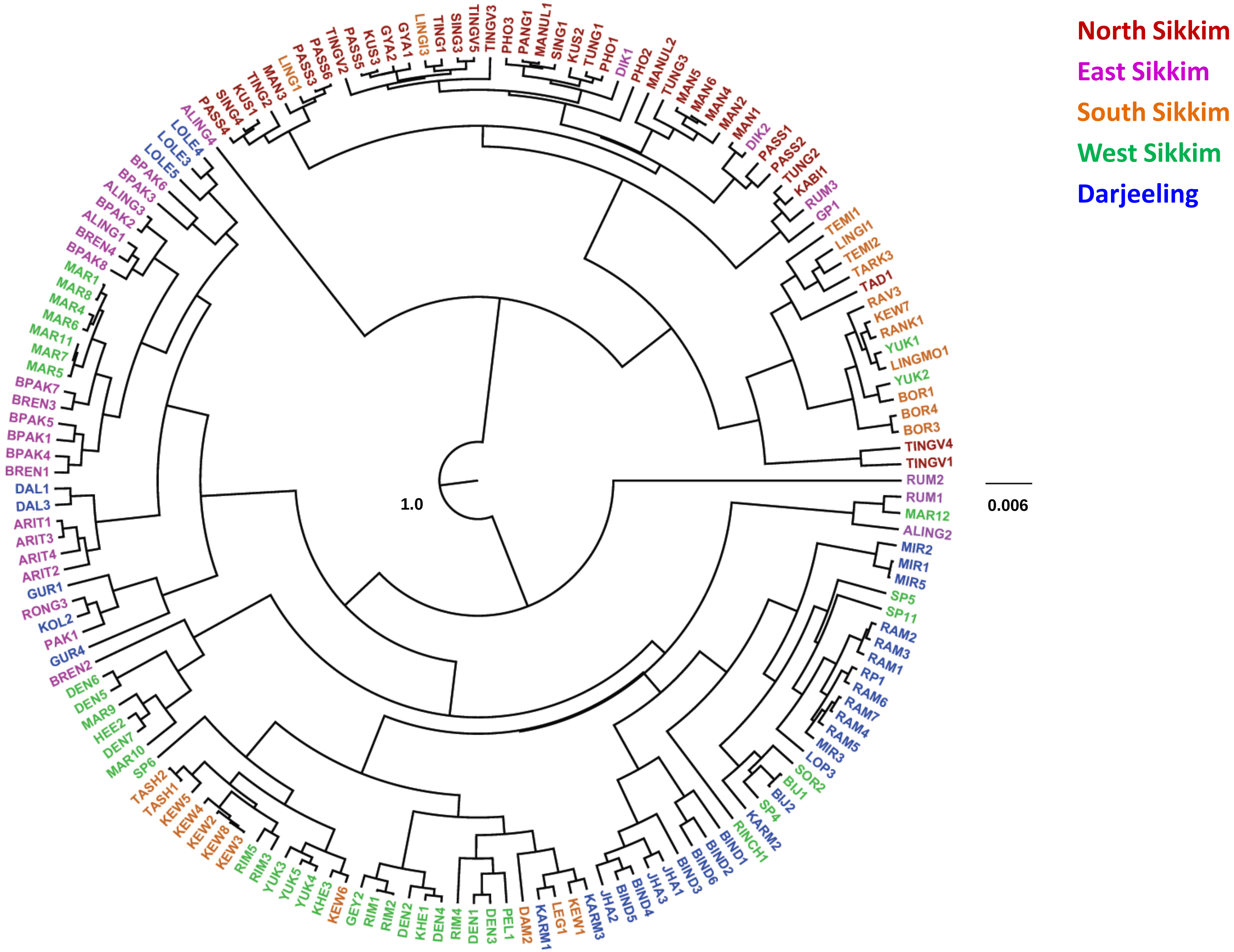

Supplementary Figure 1: DNA-R phylogeny. Isolates are coloured according to geographic districts of origin. Posterior probabilities are only displayed for clade defining nodes. Branch lengths are proportional to the scale bar, which represents the mean number of nucleotide substitutions per site.

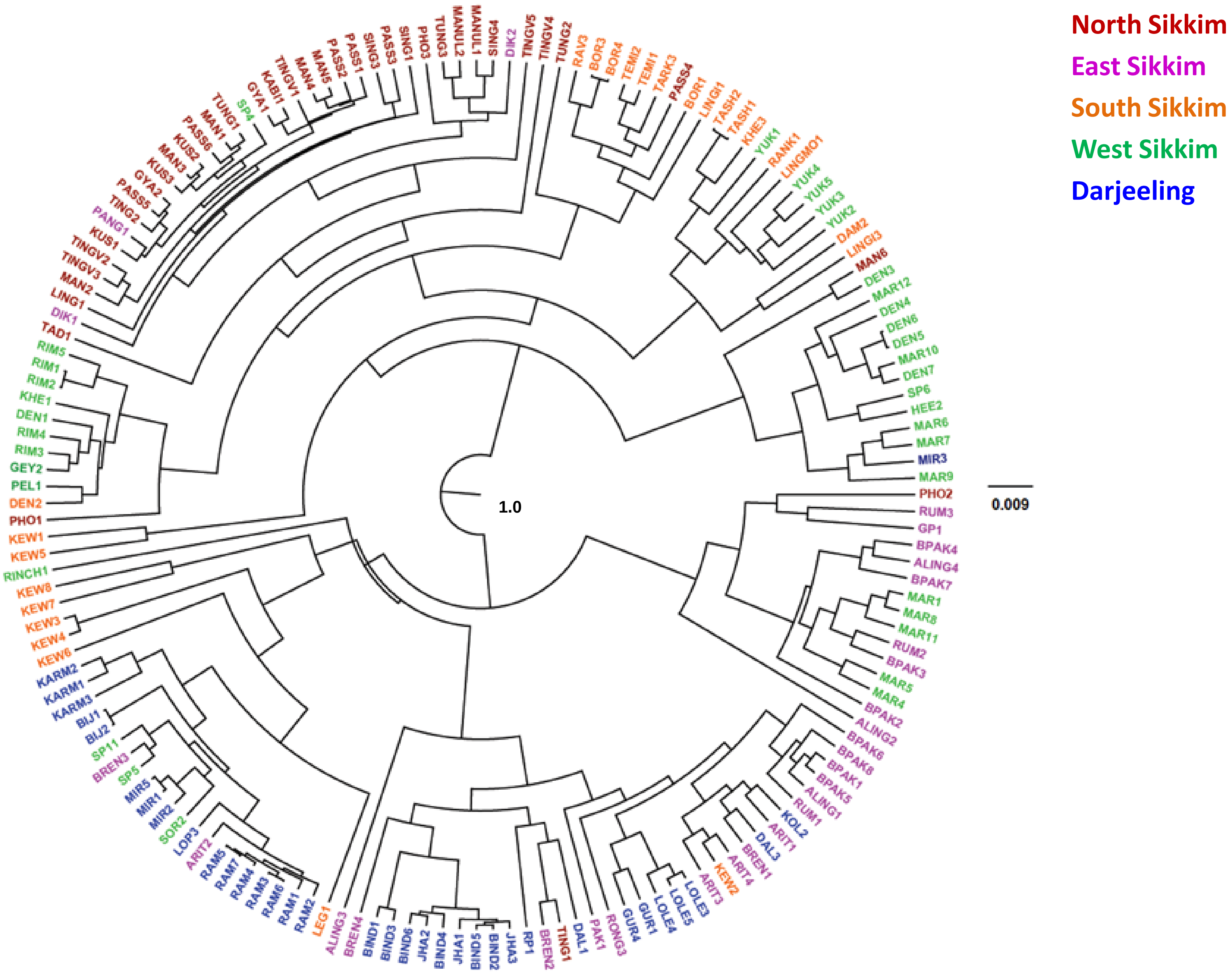

Supplementary Figure 2: DNA-U3 phylogeny. Isolates are coloured according to geographic districts of origin. Posterior probabilities are only displayed for clade defining nodes. Branch lengths are proportional to the scale bar, which represents the mean number of nucleotide substitutions per site.

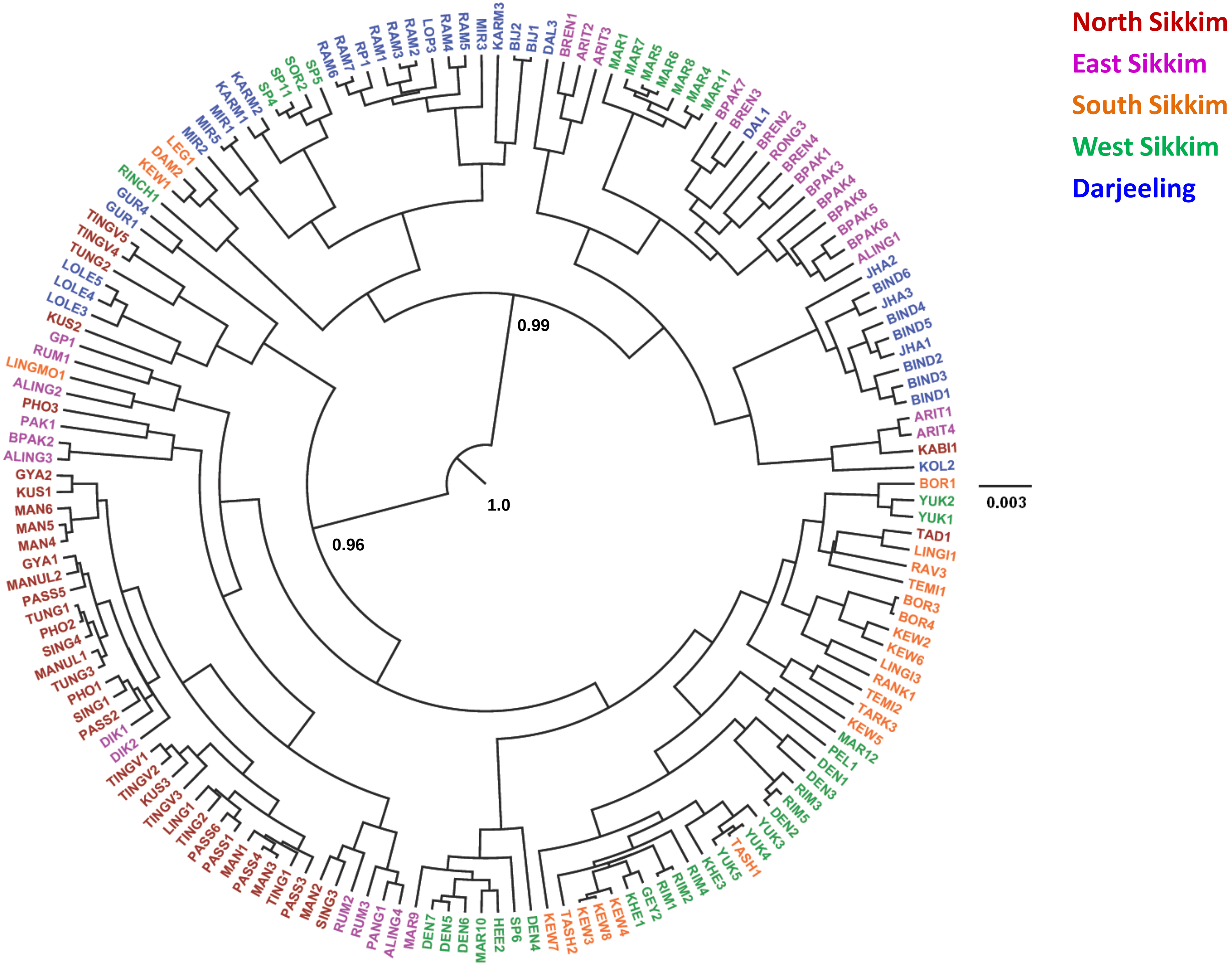

Supplementary Figure 3: DNA-S phylogeny. Isolates are coloured according to geographic districts of origin. Posterior probabilities are only displayed for clade defining nodes. Branch lengths are proportional to the scale bar, which represents the mean number of nucleotide substitutions per site.

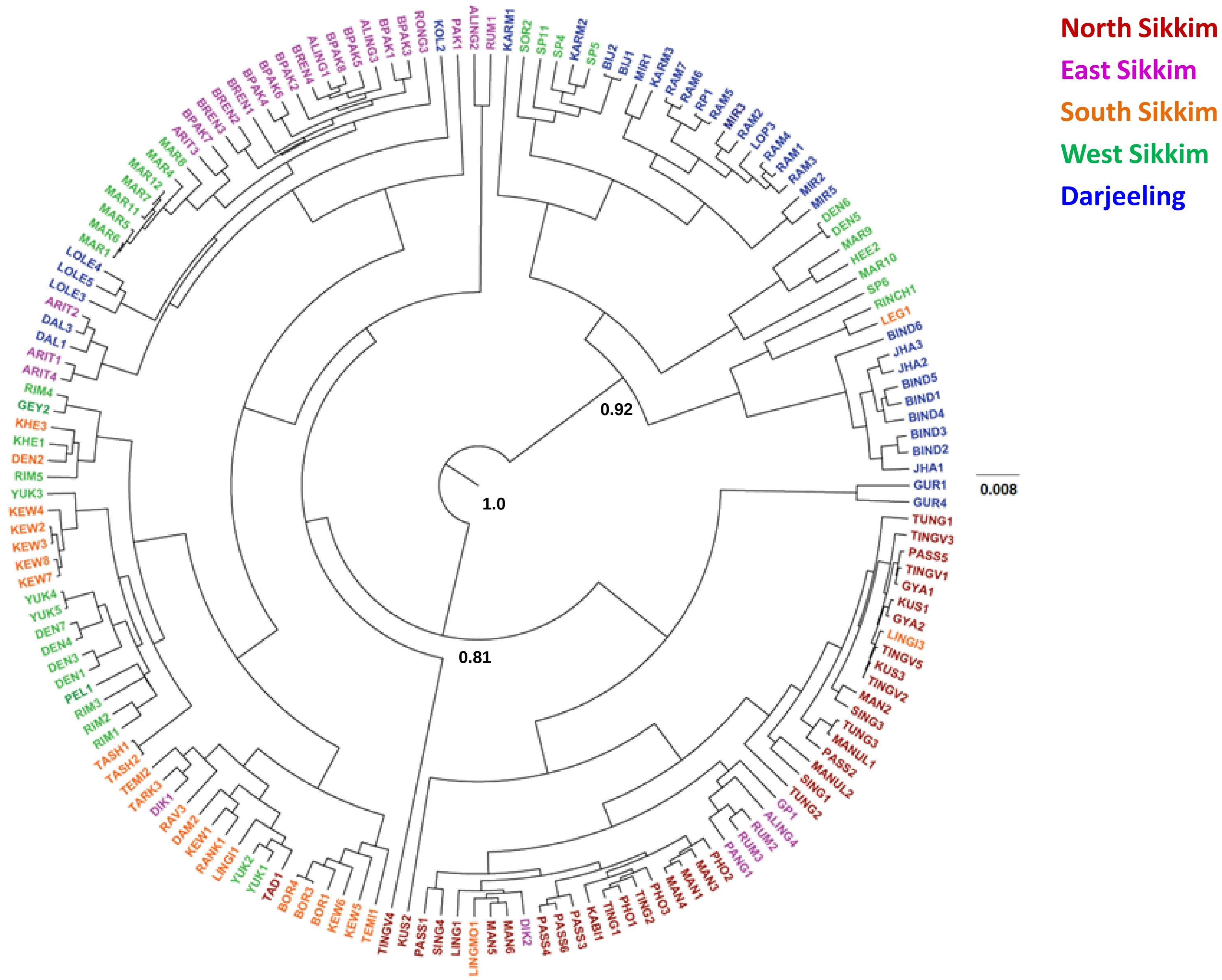

Supplementary Figure 4: DNA-M phylogeny. Isolates are coloured according to geographic districts of origin. Posterior probabilities are only displayed for clade defining nodes. Branch lengths are proportional to the scale bar, which represents the mean number of nucleotide substitutions per site.

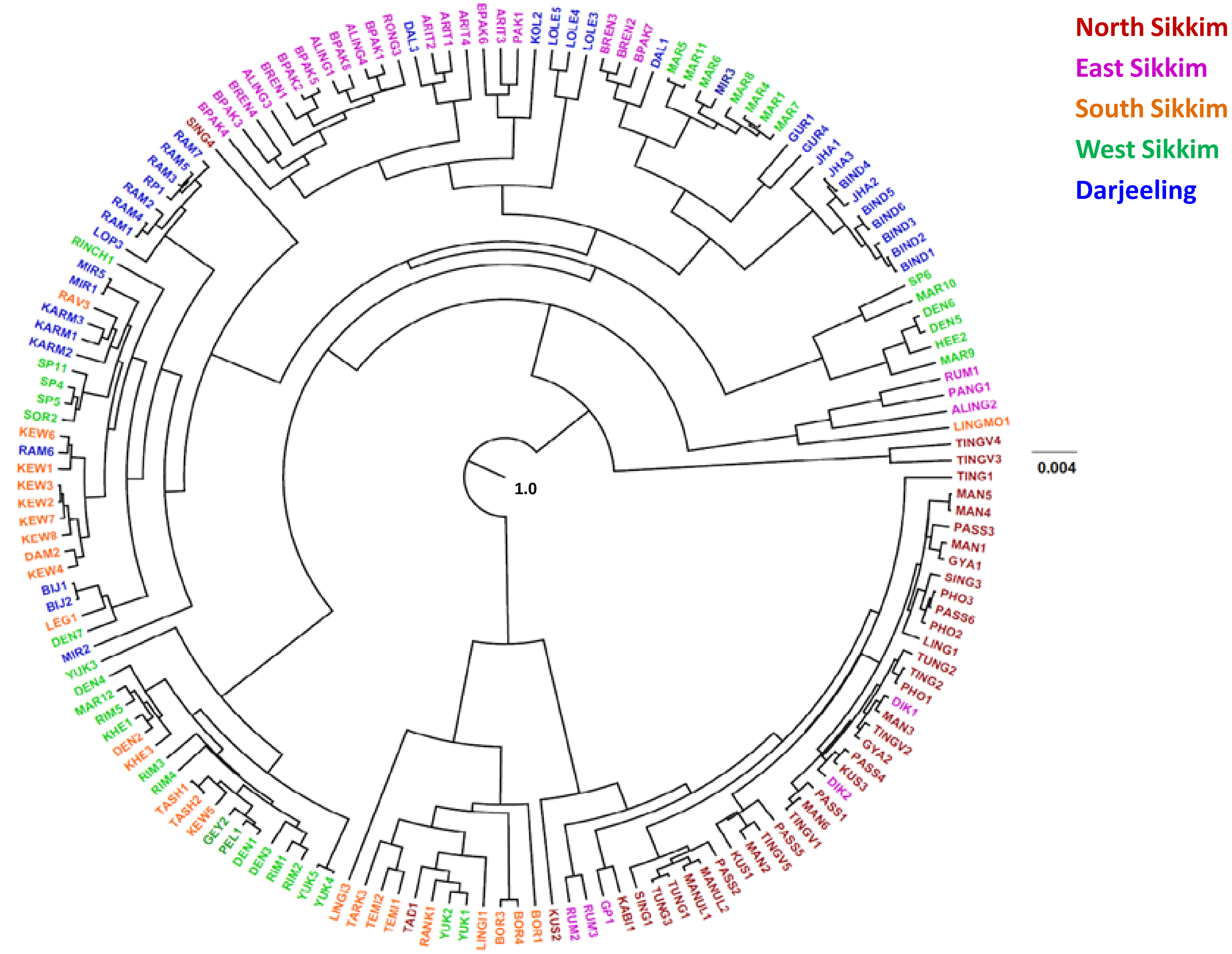

Supplementary Figure 5: DNA-C phylogeny. Isolates are coloured according to geographic districts of origin. Posterior probabilities are only displayed for clade defining nodes. Branch lengths are proportional to the scale bar, which represents the mean number of nucleotide substitutions per site.

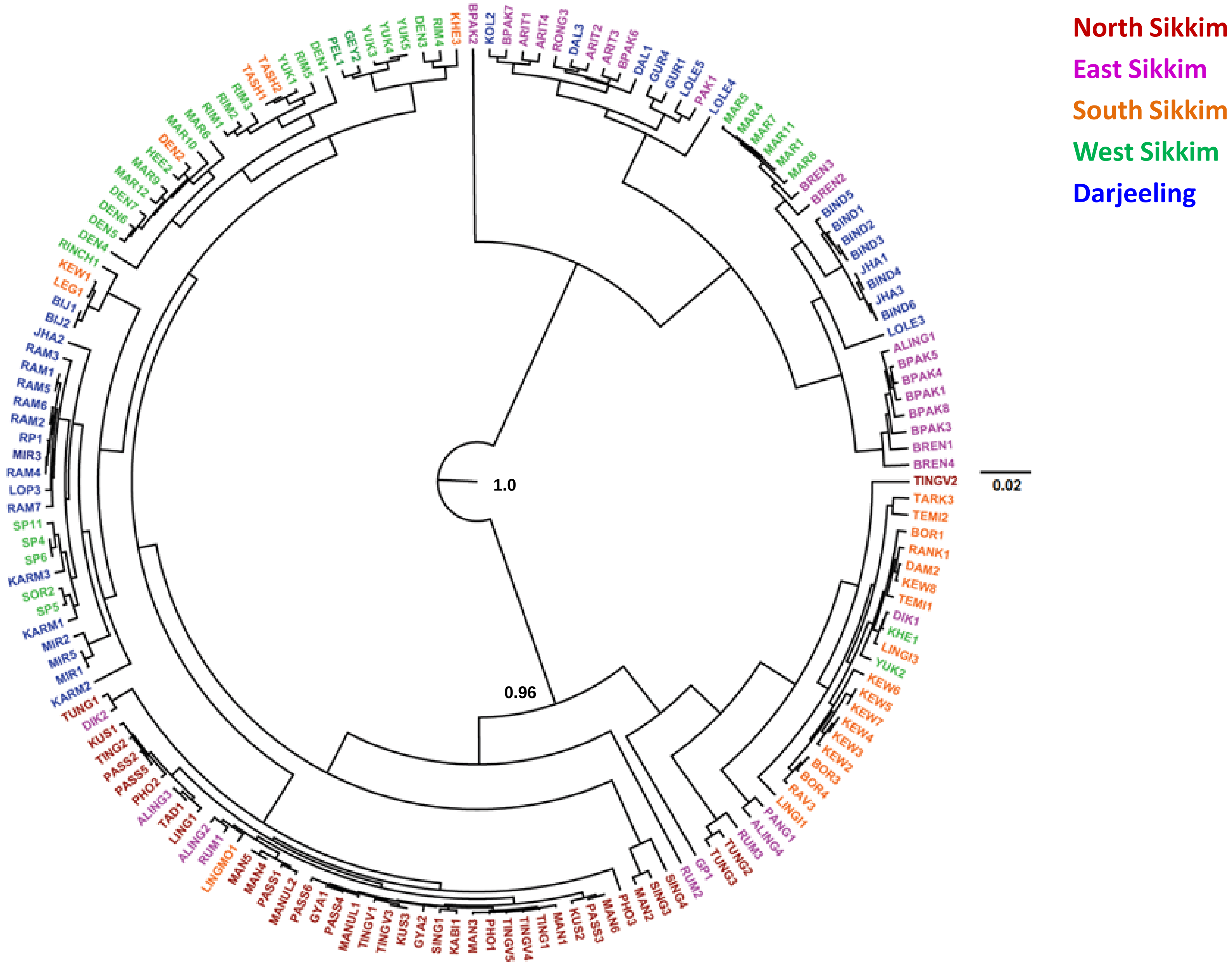

Supplementary Figure 6: DNA-N phylogeny. Isolates are coloured according to geographic districts of origin. Posterior probabilities are only displayed for clade defining nodes. Branch lengths are proportional to the scale bar, which represents the mean number of nucleotide substitutions per site.

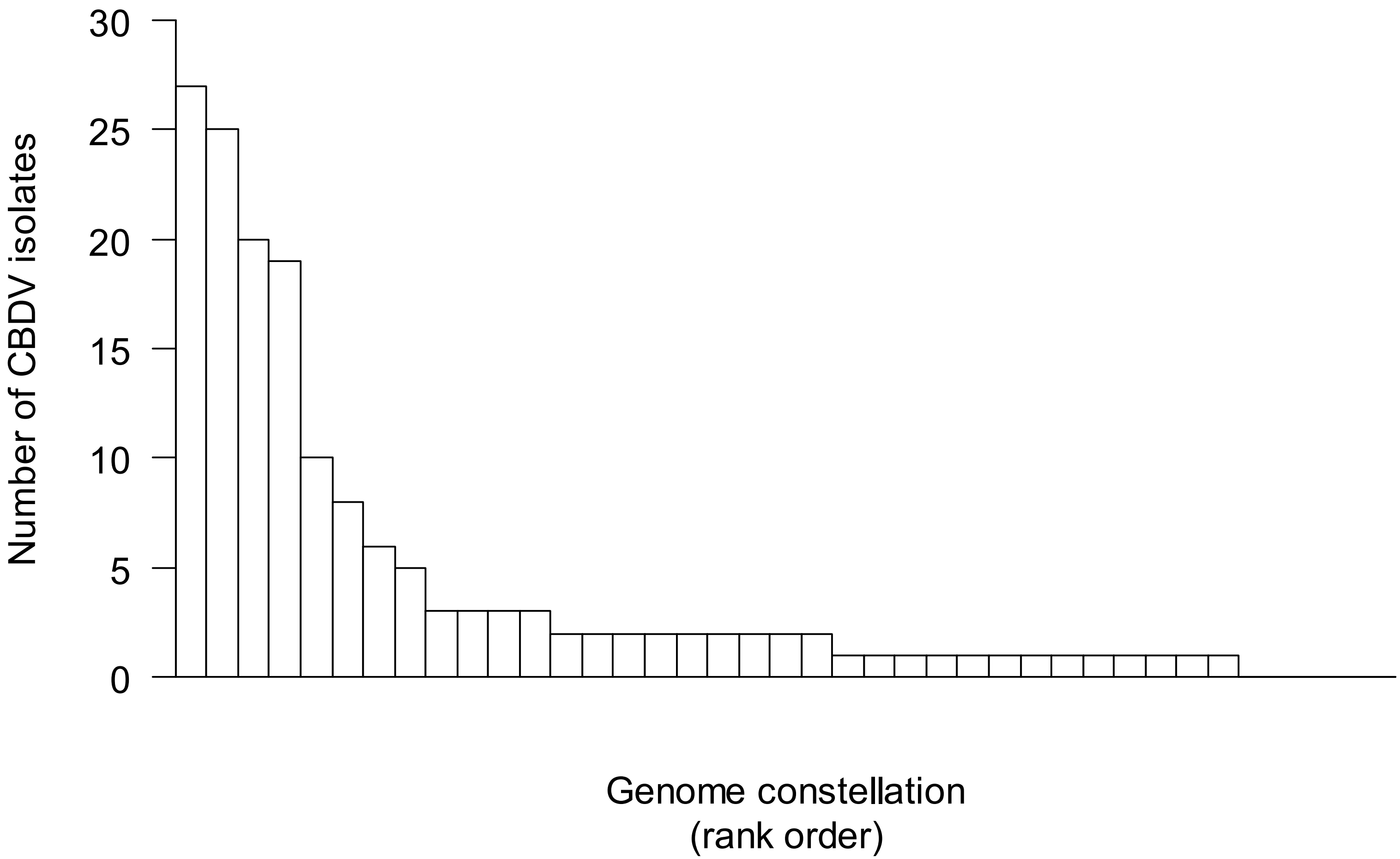

Supplementary Figure 7: Frequency distribution of unique genome constellations

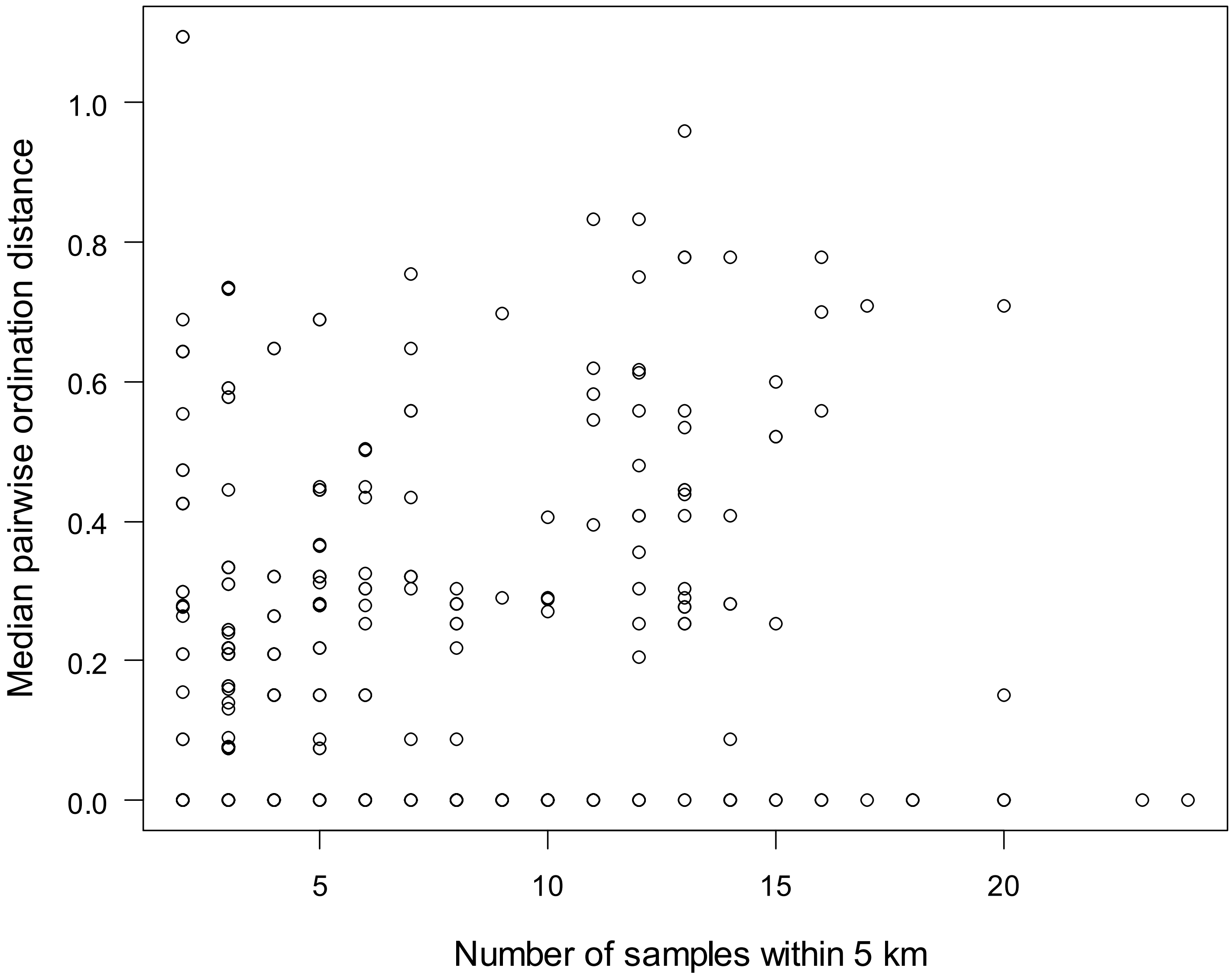

Supplementary Figure 8: Median pairwise ordination distance (genome constellation diversity) versus number of samples collected within a 5 km radius.
