# Supplementary material for: Identifying geographic hot spots of reassortment in a multipartite plant virus
Source: Evol Appl. 2014 Apr 9;7(5):569–79. doi: 10.1111/eva.12156 (PMC4055178; doi:10.1111/eva.12156)
Supplement: Supplementary file 2 [file eva0007-0569-SD2.docx]

**Supplementary Table 1**

List of CBDV isolates with corresponding districts, geographic coordinates, elevations, and Genbank accession numbers for each genome component.

| Isolate | District | Geographic coordinates (UTM) | | Elevation (m) | Genome component | | | | | |
| --- | --- | --- | --- | --- | --- | --- | --- | --- | --- | --- |
|  |  | X | Y |  | DNA-R | DNA-U3 | DNA-S | DNA-M | DNA-C | DNA-N |
| ALING1 | East Sikkim | 658549.4 | 3018308 | 972 | KF710463 | KF710626 | KF710789 | KF10952 | KF711115 | KF11278 |
| ALING2 | East Sikkim | 658655 | 3018252 | 1015 | KF710464 | KF710627 | KF710790 | KF10953 | KF711116 | KF11279 |
| ALING3 | East Sikkim | 658659.6 | 3018202 | 1015 | KF710465 | KF710628 | KF710791 | KF10954 | KF711117 | KF11280 |
| ALING4 | East Sikkim | 658656.7 | 3018201 | 1015 | KF710466 | KF710629 | KF710792 | KF10955 | KF711118 | KF11281 |
| ARIT1 | East Sikkim | 664689.2 | 3007852 | 1378 | KF710467 | KF710630 | KF710793 | KF10956 | KF711119 | KF11282 |
| ARIT2 | East Sikkim | 664694.2 | 3007852 | 1379 | KF710468 | KF710631 | KF710794 | KF10957 | KF711120 | KF11283 |
| ARIT3 | East Sikkim | 664704.2 | 3007852 | 1379 | KF710469 | KF710632 | KF710795 | KF10958 | KF711121 | KF11284 |
| ARIT4 | East Sikkim | 664704.2 | 3007862 | 1379 | KF710470 | KF710633 | KF710796 | KF10959 | KF711122 | KF11285 |
| BIJ1 | Darjeeling | 617419.1 | 2994814 | 1115 | KF710471 | KF710634 | KF710797 | KF10960 | KF711123 | KF11286 |
| BIJ2 | Darjeeling | 617422 | 2994819 | 1118 | KF710472 | KF710635 | KF710798 | KF10961 | KF711124 | KF11287 |
| BIND1 | Darjeeling | 685531 | 2999102 | 572 | KF710473 | KF710636 | KF710799 | KF10962 | KF711125 | KF11288 |
| BIND2 | Darjeeling | 685588.3 | 2998787 | 590 | KF710474 | KF710637 | KF710800 | KF10963 | KF711126 | KF11289 |
| BIND3 | Darjeeling | 685591.3 | 2998783 | 590 | KF710475 | KF710638 | KF710801 | KF10964 | KF711127 | KF11290 |
| BIND4 | Darjeeling | 685349.2 | 2996322 | 680 | KF710476 | KF710639 | KF710802 | KF10965 | KF711128 | KF11291 |
| BIND5 | Darjeeling | 685322.3 | 2996264 | 740 | KF710477 | KF710640 | KF710803 | KF10966 | KF711129 | KF11292 |
| BIND6 | Darjeeling | 685332.3 | 2996259 | 740 | KF710478 | KF710641 | KF710804 | KF10967 | KF711130 | KF11293 |
| BOR1 | South Sikkim | 632821 | 3026306 | 1729 | KF710479 | KF710642 | KF710805 | KF10968 | KF711131 | KF11294 |
| BOR3 | South Sikkim | 632739.4 | 3026259 | 1698 | KF710480 | KF710643 | KF710806 | KF10969 | KF711132 | KF11295 |
| BOR4 | South Sikkim | 632749.4 | 3026259 | 1698 | KF710481 | KF710644 | KF710807 | KF10970 | KF711133 | KF11296 |
| BPAK1 | East Sikkim | 658411.3 | 3016722 | 908 | KF710482 | KF710645 | KF710808 | KF10971 | KF711134 | KF11297 |
| BPAK2 | East Sikkim | 658442.7 | 3016745 | 908 | KF710483 | KF710646 | KF710809 | KF10972 | KF711135 | KF11298 |
| BPAK3 | East Sikkim | 658879.9 | 3015853 | 982 | KF710484 | KF710647 | KF710810 | KF10973 | KF711136 | KF11299 |
| BPAK4 | East Sikkim | 659025 | 3015351 | 1145 | KF710485 | KF710648 | KF710811 | KF10974 | KF711137 | KF11300 |
| BPAK5 | East Sikkim | 659016.1 | 3015345 | 1145 | KF710486 | KF710649 | KF710812 | KF10975 | KF711138 | KF11301 |
| BPAK6 | East Sikkim | 658480 | 3014759 | 1251 | KF710487 | KF710650 | KF710813 | KF10976 | KF711139 | KF11302 |
| BPAK7 | East Sikkim | 658471.1 | 3014753 | 1251 | KF710488 | KF710651 | KF710814 | KF10977 | KF711140 | KF11303 |
| BPAK8 | East Sikkim | 658455.4 | 3014745 | 1251 | KF710489 | KF710652 | KF710815 | KF10978 | KF711141 | KF11304 |
| BREN1 | East Sikkim | 660847.5 | 3008833 | 612 | KF710490 | KF710653 | KF710816 | KF10979 | KF711142 | KF11305 |
| BREN2 | East Sikkim | 660980.6 | 3008813 | 640 | KF710491 | KF710654 | KF710817 | KF10980 | KF711143 | KF11306 |
| BREN3 | East Sikkim | 660985.6 | 3008813 | 640 | KF710492 | KF710655 | KF710818 | KF10981 | KF711144 | KF11307 |
| BREN4 | East Sikkim | 660980.6 | 3008833 | 640 | KF710493 | KF710656 | KF710819 | KF10982 | KF711145 | KF11308 |
| DAL1 | Darjeeling | 661038.6 | 3005634 | 1036 | KF710494 | KF710657 | KF710820 | KF10983 | KF711146 | KF11309 |
| DAL3 | Darjeeling | 661737.4 | 3004409 | 776 | KF710495 | KF710658 | KF710821 | KF10984 | KF711147 | KF11310 |
| DAM2 | South Sikkim | 637097.7 | 3012931 | 2006 | KF710496 | KF710659 | KF710822 | KF10985 | KF711148 | KF11311 |
| DEN1 | West Sikkim | 617585 | 3018229 | 1768 | KF710497 | KF710660 | KF710823 | KF10986 | KF711149 | KF11312 |
| DEN2 | West Sikkim | 617587 | 3018229 | 1768 | KF710498 | KF710661 | KF710824 | KF10987 | KF711150 | KF11313 |
| DEN3 | West Sikkim | 613854.9 | 3017083 | 1585 | KF710499 | KF710662 | KF710825 | KF10988 | KF711151 | KF11314 |
| DEN4 | West Sikkim | 612282.7 | 3016226 | 1347 | KF710500 | KF710663 | KF710826 | KF10989 | KF711152 | KF11315 |
| DEN5 | West Sikkim | 612282.7 | 3016236 | 1347 | KF710501 | KF710664 | KF710827 | KF10990 | KF711153 | KF11316 |
| DEN6 | West Sikkim | 612284.7 | 3016236 | 1347 | KF710502 | KF710665 | KF710828 | KF10991 | KF711154 | KF11317 |
| DEN7 | West Sikkim | 612284.7 | 3016239 | 1347 | KF710503 | KF710666 | KF710829 | KF10992 | KF711155 | KF11318 |
| DIK1 | East Sikkim | 647804.6 | 3029026 | 889 | KF710504 | KF710667 | KF710830 | KF10993 | KF711156 | KF11319 |
| DIK2 | East Sikkim | 647814.2 | 3029052 | 840 | KF710505 | KF710668 | KF710831 | KF10994 | KF711157 | KF11320 |
| GEY2 | West Sikkim | 623181.4 | 3019864 | 1643 | KF710506 | KF710669 | KF710832 | KF10995 | KF711158 | KF11321 |
| GP1 | East Sikkim | 659935.6 | 3028762 | 1856 | KF710507 | KF710670 | KF710833 | KF10996 | KF711159 | KF11322 |
| GUR1 | Darjeeling | 667974.8 | 2992222 | 1171 | KF710508 | KF710671 | KF710834 | KF10997 | KF711160 | KF11323 |
| GUR4 | Darjeeling | 668753.3 | 2989609 | 751 | KF710509 | KF710672 | KF710835 | KF10998 | KF711161 | KF11324 |
| GYA1 | North Sikkim | 649178.8 | 3041393 | 878 | KF710510 | KF710673 | KF710836 | KF10999 | KF711162 | KF11325 |
| GYA2 | North Sikkim | 649177.6 | 3041405 | 878 | KF710511 | KF710674 | KF710837 | KF11000 | KF711163 | KF11326 |
| HEE2 | West Sikkim | 617965.9 | 3014615 | 1687 | KF710512 | KF710675 | KF710838 | KF11001 | KF711164 | KF11327 |
| JHA1 | Darjeeling | 686127.7 | 2993543 | 467 | KF710513 | KF710676 | KF710839 | KF11002 | KF711165 | KF11328 |
| JHA2 | Darjeeling | 686119 | 2993527 | 470 | KF710514 | KF710677 | KF710840 | KF11003 | KF711166 | KF11329 |
| JHA3 | Darjeeling | 686105.1 | 2993525 | 465 | KF710515 | KF710678 | KF710841 | KF11004 | KF711167 | KF11330 |
| KABI1 | North Sikkim | 660195 | 3032777 | 1493 | KF710516 | KF710679 | KF710842 | KF11005 | KF711168 | KF11331 |
| KARM1 | Darjeeling | 617605.8 | 2998784 | 1596 | KF710517 | KF710680 | KF710843 | KF11006 | KF711169 | KF11332 |
| KARM2 | Darjeeling | 617469.1 | 2998876 | 1545 | KF710518 | KF710681 | KF710844 | KF11007 | KF711170 | KF11333 |
| KARM3 | Darjeeling | 617461.2 | 2998871 | 1520 | KF710519 | KF710682 | KF710845 | KF11008 | KF711171 | KF11334 |
| KEW1 | South Sikkim | 629353.2 | 3018495 | 1447 | KF710520 | KF710683 | KF710846 | KF11009 | KF711172 | KF11335 |
| KEW2 | South Sikkim | 629354.2 | 3018495 | 1447 | KF710521 | KF710684 | KF710847 | KF11010 | KF711173 | KF11336 |
| KEW3 | South Sikkim | 629361.2 | 3018495 | 1447 | KF710522 | KF710685 | KF710848 | KF11011 | KF711174 | KF11337 |
| KEW4 | South Sikkim | 629365.2 | 3018495 | 1447 | KF710523 | KF710686 | KF710849 | KF11012 | KF711175 | KF11338 |
| KEW5 | South Sikkim | 629373.2 | 3018495 | 1447 | KF710524 | KF710687 | KF710850 | KF11013 | KF711176 | KF11339 |
| KEW6 | South Sikkim | 629353.2 | 3018515 | 1447 | KF710525 | KF710688 | KF710851 | KF11014 | KF711177 | KF11340 |
| KEW7 | South Sikkim | 629356.2 | 3018515 | 1447 | KF710526 | KF710689 | KF710852 | KF11015 | KF711178 | KF11341 |
| KEW8 | South Sikkim | 629360.2 | 3018515 | 1447 | KF710527 | KF710690 | KF710853 | KF11016 | KF711179 | KF11342 |
| KHE1 | West Sikkim | 620218.8 | 3023858 | 1360 | KF710528 | KF710691 | KF710854 | KF11017 | KF711180 | KF11343 |
| KHE3 | West Sikkim | 617880.2 | 3024534 | 1608 | KF710529 | KF710692 | KF710855 | KF11018 | KF711181 | KF11344 |
| KOL2 | Darjeeling | 666494.8 | 2998963 | 1738 | KF710530 | KF710693 | KF710856 | KF11019 | KF711182 | KF11345 |
| KUS1 | North Sikkim | 645711 | 3049136 | 1619 | KF710531 | KF710694 | KF710857 | KF11020 | KF711183 | KF11346 |
| KUS2 | North Sikkim | 645707.5 | 3049093 | 1619 | KF710532 | KF710695 | KF710858 | KF11021 | KF711184 | KF11347 |
| KUS3 | North Sikkim | 646305.7 | 3048953 | 1679 | KF710533 | KF710696 | KF710859 | KF11022 | KF711185 | KF11348 |
| LEG1 | South Sikkim | 625401.6 | 3018586 | 956 | KF710534 | KF710697 | KF710860 | KF11023 | KF711186 | KF11349 |
| LING1 | North Sikkim | 650269.5 | 3043101 | 936 | KF710535 | KF710698 | KF710861 | KF11024 | KF711187 | KF11350 |
| LINGI1 | South Sikkim | 645328 | 3027413 | 870 | KF710536 | KF710699 | KF710862 | KF11025 | KF711188 | KF11351 |
| LINGI3 | South Sikkim | 645456.1 | 3026707 | 781 | KF710537 | KF710700 | KF710863 | KF11026 | KF711189 | KF11352 |
| LINGMO1 | South Sikkim | 646286 | 3024049 | 600 | KF710538 | KF710701 | KF710864 | KF11027 | KF711190 | KF11353 |
| LOLE3 | Darjeeling | 655422.4 | 2989693 | 1682 | KF710539 | KF710702 | KF710865 | KF11028 | KF711191 | KF11354 |
| LOLE4 | Darjeeling | 655404.1 | 2989735 | 1663 | KF710540 | KF710703 | KF710866 | KF11029 | KF711192 | KF11355 |
| LOLE5 | Darjeeling | 655420.3 | 2989704 | 1682 | KF710541 | KF710704 | KF710867 | KF11030 | KF711193 | KF11356 |
| LOP3 | Darjeeling | 636695 | 2993951 | 1418 | KF710542 | KF710705 | KF710868 | KF11031 | KF711194 | KF11357 |
| MAN1 | North Sikkim | 652017 | 3043162 | 1204 | KF710543 | KF710706 | KF710869 | KF11032 | KF711195 | KF11358 |
| MAN2 | North Sikkim | 652019 | 3043162 | 1204 | KF710544 | KF710707 | KF710870 | KF11033 | KF711196 | KF11359 |
| MAN3 | North Sikkim | 652037 | 3043162 | 1229 | KF710545 | KF710708 | KF710871 | KF11034 | KF711197 | KF11360 |
| MAN4 | North Sikkim | 652037 | 3043177 | 1229 | KF710546 | KF710709 | KF710872 | KF11035 | KF711198 | KF11361 |
| MAN5 | North Sikkim | 652037 | 3043192 | 1229 | KF710547 | KF710710 | KF710873 | KF11036 | KF711199 | KF11362 |
| MAN6 | North Sikkim | 652047 | 3043192 | 1229 | KF710548 | KF710711 | KF710874 | KF11037 | KF711200 | KF11363 |
| MANUL1 | North Sikkim | 655795.9 | 3044332 | 1488 | KF710549 | KF710712 | KF710875 | KF11038 | KF711201 | KF11364 |
| MANUL2 | North Sikkim | 655821.6 | 3044334 | 1472 | KF710550 | KF710713 | KF710876 | KF11039 | KF711202 | KF11365 |
| MAR1 | West Sikkim | 620867 | 3015037 | 1508 | KF710551 | KF710714 | KF710877 | KF11040 | KF711203 | KF11366 |
| MAR4 | West Sikkim | 620779.6 | 3014756 | 1581 | KF710552 | KF710715 | KF710878 | KF11041 | KF711204 | KF11367 |
| MAR5 | West Sikkim | 620936.2 | 3014742 | 1591 | KF710553 | KF710716 | KF710879 | KF11042 | KF711205 | KF11368 |
| MAR6 | West Sikkim | 621003.6 | 3014633 | 1628 | KF710554 | KF710717 | KF710880 | KF11043 | KF711206 | KF11369 |
| MAR7 | West Sikkim | 620823.4 | 3015035 | 1501 | KF710555 | KF710718 | KF710881 | KF11044 | KF711207 | KF11370 |
| MAR8 | West Sikkim | 620846.3 | 3015030 | 1501 | KF710556 | KF710719 | KF710882 | KF11045 | KF711208 | KF11371 |
| MAR9 | West Sikkim | 620814.4 | 3014945 | 1529 | KF710557 | KF710720 | KF710883 | KF11046 | KF711209 | KF11372 |
| MAR10 | West Sikkim | 620773.8 | 3014952 | 1532 | KF710558 | KF710721 | KF710884 | KF11047 | KF711210 | KF11373 |
| MAR11 | West Sikkim | 620823.1 | 3014972 | 1529 | KF710559 | KF710722 | KF710885 | KF11048 | KF711211 | KF11374 |
| MAR12 | West Sikkim | 620701.7 | 3014827 | 1560 | KF710560 | KF710723 | KF710886 | KF11049 | KF711212 | KF11375 |
| MIR1 | Darjeeling | 615143.8 | 2976373 | 1683 | KF710561 | KF710724 | KF710887 | KF11050 | KF711213 | KF11376 |
| MIR2 | Darjeeling | 615150.8 | 2976373 | 1683 | KF710562 | KF710725 | KF710888 | KF11051 | KF711214 | KF11377 |
| MIR3 | Darjeeling | 615155.8 | 2976373 | 1683 | KF710563 | KF710726 | KF710889 | KF11052 | KF711215 | KF11378 |
| MIR5 | Darjeeling | 615143.8 | 2976393 | 1683 | KF710564 | KF710727 | KF710890 | KF11053 | KF711216 | KF11379 |
| PAK1 | East Sikkim | 657751.8 | 3013299 | 1307 | KF710565 | KF710728 | KF710891 | KF11054 | KF711217 | KF11380 |
| PANG1 | East Sikkim | 656519.8 | 3028526 | 1935 | KF710566 | KF710729 | KF710892 | KF11055 | KF711218 | KF11381 |
| PASS1 | North Sikkim | 649616.3 | 3045857 | 886 | KF710567 | KF710730 | KF710893 | KF11056 | KF711219 | KF11382 |
| PASS2 | North Sikkim | 649623.3 | 3045854 | 886 | KF710568 | KF710731 | KF710894 | KF11057 | KF711220 | KF11383 |
| PASS3 | North Sikkim | 649620.1 | 3045875 | 873 | KF710569 | KF710732 | KF710895 | KF11058 | KF711221 | KF11384 |
| PASS4 | North Sikkim | 649613.1 | 3045879 | 873 | KF710570 | KF710733 | KF710896 | KF11059 | KF711222 | KF11385 |
| PASS5 | North Sikkim | 649200.6 | 3046331 | 872 | KF710571 | KF710734 | KF710897 | KF11060 | KF711223 | KF11386 |
| PASS6 | North Sikkim | 649206.7 | 3046318 | 951 | KF710572 | KF710735 | KF710898 | KF11061 | KF711224 | KF11387 |
| PEL1 | West Sikkim | 619931 | 3020680 | 1766 | KF710573 | KF710736 | KF710899 | KF11062 | KF711225 | KF11388 |
| PHO1 | North Sikkim | 656665.4 | 3034418 | 1718 | KF710574 | KF710737 | KF710900 | KF11063 | KF711226 | KF11389 |
| PHO2 | North Sikkim | 656669.3 | 3034425 | 1718 | KF710575 | KF710738 | KF710901 | KF11064 | KF711227 | KF11390 |
| PHO3 | North Sikkim | 656695.4 | 3034418 | 1703 | KF710576 | KF710739 | KF710902 | KF11065 | KF711228 | KF11391 |
| RAM1 | Darjeeling | 629958.2 | 2988024 | 1687 | KF710577 | KF710740 | KF710903 | KF11066 | KF711229 | KF11392 |
| RAM2 | Darjeeling | 629960.2 | 2988024 | 1687 | KF710578 | KF710741 | KF710904 | KF11067 | KF711230 | KF11393 |
| RAM3 | Darjeeling | 629970.2 | 2988024 | 1661 | KF710579 | KF710742 | KF710905 | KF11068 | KF711231 | KF11394 |
| RAM4 | Darjeeling | 629988.6 | 2988062 | 1678 | KF710580 | KF710743 | KF710906 | KF11069 | KF711232 | KF11395 |
| RAM5 | Darjeeling | 629976.5 | 2988075 | 1678 | KF710581 | KF710744 | KF710907 | KF11070 | KF711233 | KF11396 |
| RAM6 | Darjeeling | 629991.3 | 2988082 | 1678 | KF710582 | KF710745 | KF710908 | KF11071 | KF711234 | KF11397 |
| RAM7 | Darjeeling | 630042.4 | 2988040 | 1678 | KF710583 | KF710746 | KF710909 | KF11072 | KF711235 | KF11398 |
| RANK1 | South Sikkim | 636513.1 | 3016280 | 1580 | KF710584 | KF710747 | KF710910 | KF11073 | KF711236 | KF11399 |
| RAV3 | South Sikkim | 631887.3 | 3018043 | 1779 | KF710585 | KF710748 | KF710911 | KF11074 | KF711237 | KF11400 |
| RIM1 | West Sikkim | 617623 | 3021422 | 1461 | KF710586 | KF710749 | KF710912 | KF11075 | KF711238 | KF11401 |
| RIM2 | West Sikkim | 617624 | 3021422 | 1461 | KF710587 | KF710750 | KF710913 | KF11076 | KF711239 | KF11402 |
| RIM3 | West Sikkim | 617914.9 | 3021830 | 1267 | KF710588 | KF710751 | KF710914 | KF11077 | KF711240 | KF11403 |
| RIM4 | West Sikkim | 617924.9 | 3021830 | 1267 | KF710589 | KF710752 | KF710915 | KF11078 | KF711241 | KF11404 |
| RIM5 | West Sikkim | 617925.9 | 3021830 | 1267 | KF710590 | KF710753 | KF710916 | KF11079 | KF711242 | KF11405 |
| RINCH1 | West Sikkim | 626231.1 | 3013443 | 1594 | KF710591 | KF710754 | KF710917 | KF11080 | KF711243 | KF11406 |
| RONG3 | East Sikkim | 666218.1 | 3010459 | 723 | KF710592 | KF710755 | KF710918 | KF11081 | KF711244 | KF11407 |
| RP1 | Darjeeling | 629890.2 | 2988073 | 1704 | KF710593 | KF710756 | KF710919 | KF11082 | KF711245 | KF11408 |
| RUM1 | East Sikkim | 656423.9 | 3020792 | 1079 | KF710594 | KF710757 | KF710920 | KF11083 | KF711246 | KF11409 |
| RUM2 | East Sikkim | 656463.5 | 3020792 | 1042 | KF710595 | KF710758 | KF710921 | KF11084 | KF711247 | KF11410 |
| RUM3 | East Sikkim | 655213.1 | 3021281 | 1320 | KF710596 | KF710759 | KF710922 | KF11085 | KF711248 | KF11411 |
| SING1 | North Sikkim | 653232.9 | 3044587 | 1342 | KF710597 | KF710760 | KF710923 | KF11086 | KF711249 | KF11412 |
| SING3 | North Sikkim | 653232.9 | 3044602 | 1342 | KF710598 | KF710761 | KF710924 | KF11087 | KF711250 | KF11413 |
| SING4 | North Sikkim | 653237.9 | 3044602 | 1342 | KF710599 | KF710762 | KF710925 | KF11088 | KF711251 | KF11414 |
| SOR2 | West Sikkim | 619157.3 | 3006348 | 1666 | KF710600 | KF710763 | KF710926 | KF11089 | KF711252 | KF11415 |
| SP11 | West Sikkim | 616841.8 | 3004751 | 1598 | KF710601 | KF710764 | KF710927 | KF11090 | KF711253 | KF11416 |
| SP4 | West Sikkim | 616912.1 | 3004758 | 1610 | KF710602 | KF710765 | KF710928 | KF11091 | KF711254 | KF11417 |
| SP5 | West Sikkim | 616847.7 | 3004762 | 1580 | KF710603 | KF710766 | KF710929 | KF11092 | KF711255 | KF11418 |
| SP6 | West Sikkim | 617011.1 | 3004769 | 1592 | KF710604 | KF710767 | KF710930 | KF11093 | KF711256 | KF11419 |
| TAD1 | North Sikkim | 648920.8 | 3037082 | 880 | KF710605 | KF710768 | KF710931 | KF11094 | KF711257 | KF11420 |
| TARK3 | South Sikkim | 638194.5 | 3014575 | 1244 | KF710606 | KF710769 | KF710932 | KF11095 | KF711258 | KF11421 |
| TASH1 | South Sikkim | 628355.8 | 3019693 | 729 | KF710607 | KF710770 | KF710933 | KF11096 | KF711259 | KF11422 |
| TASH2 | South Sikkim | 628357.8 | 3019693 | 729 | KF710608 | KF710771 | KF710934 | KF11097 | KF711260 | KF11423 |
| TEMI1 | South Sikkim | 639814.4 | 3012484 | 1995 | KF710609 | KF710772 | KF710935 | KF11098 | KF711261 | KF11424 |
| TEMI2 | South Sikkim | 639982.2 | 3012536 | 1939 | KF710610 | KF710773 | KF710936 | KF11099 | KF711262 | KF11425 |
| TING1 | North Sikkim | 650817.9 | 3038442 | 1057 | KF710611 | KF710774 | KF710937 | KF11100 | KF711263 | KF11426 |
| TING2 | North Sikkim | 650822.7 | 3038448 | 1057 | KF710612 | KF710775 | KF710938 | KF11101 | KF711264 | KF11427 |
| TINGV1 | North Sikkim | 645966.8 | 3047308 | 1075 | KF710613 | KF710776 | KF710939 | KF11102 | KF711265 | KF11428 |
| TINGV2 | North Sikkim | 645985.3 | 3047334 | 1099 | KF710614 | KF710777 | KF710940 | KF11103 | KF711266 | KF11429 |
| TINGV3 | North Sikkim | 645936.2 | 3047390 | 1075 | KF710615 | KF710778 | KF710941 | KF11104 | KF711267 | KF11430 |
| TINGV4 | North Sikkim | 645858.4 | 3047957 | 1249 | KF710616 | KF710779 | KF710942 | KF11105 | KF711268 | KF11431 |
| TINGV5 | North Sikkim | 645797 | 3047964 | 1212 | KF710617 | KF710780 | KF710943 | KF11106 | KF711269 | KF11432 |
| TUNG1 | North Sikkim | 659448.1 | 3046868 | 1445 | KF710618 | KF710781 | KF710944 | KF11107 | KF711270 | KF11433 |
| TUNG2 | North Sikkim | 659570.4 | 3047028 | 1402 | KF710619 | KF710782 | KF710945 | KF11108 | KF711271 | KF11434 |
| TUNG3 | North Sikkim | 659594.1 | 3047034 | 1412 | KF710620 | KF710783 | KF710946 | KF11109 | KF711272 | KF11435 |
| YUK1 | West Sikkim | 620144.7 | 3027398 | 1543 | KF710621 | KF710784 | KF710947 | KF11110 | KF711273 | KF11436 |
| YUK2 | West Sikkim | 620525.8 | 3027269 | 1692 | KF710622 | KF710785 | KF710948 | KF11111 | KF711274 | KF11437 |
| YUK3 | West Sikkim | 621273.2 | 3026814 | 1425 | KF710623 | KF710786 | KF710949 | KF11112 | KF711275 | KF11438 |
| YUK4 | West Sikkim | 621403.1 | 3026774 | 1435 | KF710624 | KF710787 | KF710950 | KF11113 | KF711276 | KF11439 |
| YUK5 | West Sikkim | 621394.2 | 3026777 | 1400 | KF710625 | KF710788 | KF710951 | KF11114 | KF711277 | KF11440 |

**Supplementary Table 2**

Genetic distances within and between clades for each genome component

| Genome component | | DNA-R | DNA-U3 | DNA-S | DNA-M | DNA-C | DNA-N |
| --- | --- | --- | --- | --- | --- | --- | --- |
| Between group distance* | Major clades | 0.019 | 0.043 | 0.026 | 0.043 | 0.019 | 0.064 |
|  | Minor clades (1) | n/a | n/a | 0.013 | 0.025 | n/a | 0.083 |
|  | Minor clades (2) | n/a | n/a | 0.017 | 0.033 | n/a | n/a |
| Within group distance* | Major clade 1 | 0.008 | 0.029 | 0.012 | 0.021 | 0.011 | 0.036 |
|  | Major clade 2 | 0.01 | 0.029 | 0.015 | 0.020 | 0.013 | 0.019 |
|  | Minor clade 1.1 | n/a | n/a | 0.012 | 0.012 | n/a | 0.021 |
|  | Minor clade 1.2 | n/a | n/a | 0.009 | 0.019 | n/a | 0.009 |
|  | Minor clade 2.1 | n/a | n/a | 0.012 | 0.013 | n/a | n/a |
|  | Minor clade 2.2 | n/a | n/a | 0.010 | 0.025 | n/a | n/a |

*Distances represent the mean number of pairwise nucleotide substitutions per site (Nei, 1987)

Numbers in parenthesis correspond to the major clades within which the minor clades belong.
